# Preoperative Prevalence of *Staphylococcus aureus* in Cardiothoracic and Neurological Surgical Patients

**DOI:** 10.3389/fpubh.2014.00204

**Published:** 2014-11-03

**Authors:** Ritu Kapoor, Christopher J. Barnett, Rebecca M. Gutmann, Vedat O. Yildiz, Nicholas C. Joseph, Nicoleta Stoicea, Stephan Reyes, Barbara M. Rogers

**Affiliations:** ^1^Department of Anesthesiology, Wexner Medical Center, The Ohio State University, Columbus, OH, USA; ^2^Temple University School of Medicine, Philadelphia, PA, USA; ^3^Department of Biomedical Informatics, Wexner Medical Center, The Ohio State University, Columbus, OH, USA; ^4^The Ohio State University, Columbus, OH, USA

**Keywords:** methicillin-resistant *Staphylococcus aureus*, methicillin-susceptible *Staphylococcus aureus*, community-acquired infections, nosocomial, surgical-site infection, neurological surgery, cardiothoracic surgery, preoperative screening

## Abstract

Methicillin-resistant *Staphylococcus aureus* (MRSA) is a global cause of both hospital and community-acquired infection. This retrospective, observational study determined the prevalence of MRSA carriers in cardiothoracic and neurological surgical patients presenting to an outpatient preoperative assessment center in Columbus, OH. Aggressive skin and soft-tissue infection may be caused by MRSA with potentially fatal complications. Cardiothoracic and neurological surgical patients are at high risk for surgical-site infection. Results indicated that 4.25% of the sample carried MRSA and 25.25% carried methicillin-sensitive *S. aureus*.

## Introduction

Increasing reports of antibiotic-resistant bacterial infection and hospital-acquired infection suggest a need for improved hospital surveillance of pathogenic bacteria. In particular, *Staphylococcus aureus* (SA) deserves monitoring because of its virulence and prevalence and because strains of methicillin-resistant SA (MRSA) constitute a worldwide cause of both hospital and community-acquired infection (1, 2).

Estimates report that between 30 and 50% of adults are colonized with SA during their lifetimes, and that these apparently healthy carriers are responsible for much of the spread of the bacterium (3). There is no genetic difference between commensal and virulent forms of SA, so carriers are at risk of transmitting the pathogen to others and becoming infected themselves through broken skin or mucosal barriers (3, 4). Adhesion-receptor interactions allow for the spread of SA within the epithelium and endothelium (3, 5, 6). Once bound to the host cell, SA is phagocytized, limiting immune surveillance and certain antibiotic mechanisms (3). Heptameric alpha-hemolysin toxin forms transmembrane pores in the host cell’s wall, and subsequent cell lysis releases cell byproducts that trigger inflammatory cytokines (3, 7). Depending on the site of infection, damage can result to cardiac, vascular, neurological, respiratory, or other tissues (3). Untreated, SA can be fatal (e.g., by causing pneumonia or sepsis) with the overall SA mortality rate between 11 and 43% (3, 8).

In recent years, the proportion of MRSA infections to methicillin-sensitive SA (MSSA) infections has substantially increased (9). MRSA are strains of SA expressing the staphylococcal cassette chromosome *mec* (SCC*mec*), a mobile genetic element conferring resistance to methicillin and related beta-lactam class antimicrobials via production of a mutated penicillin binding protein (PBP) that has reduced drug binding affinity (10). Due to the genetic mobility of the resistance factor, MRSA can be contracted from contact with existing colonies and can be developed *de novo* in individuals already carrying an SA genotype (11). Individuals infected with MRSA are more difficult to treat than those with MSSA, and experience extended hospital stays, worsened clinical outcomes, and larger hospital bills (2, 12, 13). Specifically, head and neck surgical patients infected with MRSA had their hospital stays increased three-fold compared to non-infected patients and incurred increased antibiotic costs of approximately $3810 (12). Moreover, MRSA-induced soft-tissue lesions create opportunistic environments for subsequent MRSA infection, complicating treatment (14).

Methicillin-resistant SA was previously thought to be primarily hospital-acquired, with risk factors including prior hospitalization within the past 12 months, longer length of stay, a history of surgery, and use of macrolide or levofloxacin (2, 15, 16). MRSA transmission has become increasingly common in community populations as well (9, 11, 17, 18). Risk factors for community-acquired MRSA include homelessness, IV drug use, incarceration (2, 15, 19, 20) and skin or soft-tissue infection, particularly if associated with immunodeficiency (19, 21). Nevertheless, community-acquired MRSA is now commonly seen in individuals lacking any known risk factors, making monitoring, and prevention more challenging (18).

Several reports suggest early identification of incoming patient carriers through improved MRSA screening. A trial of universal screening concluded that 64% of infected patients were unknowingly carrying MRSA (10). Compared to universal screening, targeted screening may be preferable because it incurs lower hospital costs without significantly undermining surveillance quality (2, 22, 23). Ahmad et al. (2) analyzed the prevalence of MRSA infection within an almost-universal screening regimen (75%) compared to 25% screening. The study observed a decrease in MRSA detection from 2.3 to 2% and inferred that targeted screening is a sufficient alternative to universal screening.

The nares are a particularly common area for MRSA colonization, and screening of the nares accounts for up to73% of carrier detections (1). Identifying carriers early is vital for patients undergoing surgery, as MRSA is a leading cause of surgical-site infection (SSI) (22, 24). Patients undergoing cardiothoracic and neurological surgery are at an elevated risk of developing SSIs due to endogenous and hematogenous pathogen access to the thoracic and cranial cavities, with SSIs from MRSA strongly correlating with postoperative morbidity and mortality (22, 24–26). In spite of these risks, cardiothoracic and neurological surgery patients appear to be at a low risk for contracting MRSA. Jog et al. (22) reported a MRSA prevalence of only 2.5% in cardiothoracic surgical patients within the United Kingdom. Such low reported prevalence calls into question the need for preoperative screening in these patient populations. This study documents the prevalence of patients presenting preoperatively with community-acquired MRSA prior to cardiothoracic or neurological surgery at a large, urban research hospital. The purpose of the study was to determine whether this population should be omitted from selective MRSA screening protocols due to a low reported prevalence of MRSA.

## Materials and Methods

A retrospective, non-randomized, observational study was conducted in patients 18 years of age or older presenting to an outpatient preoperative assessment center for cardiothoracic or neurological surgery at a large, urban research hospital.

During the preoperative assessment, a nasal swab test was performed. Ames red dual swab kits were used to collect nasal swabs from patients upon admission to the hospital. One swab was collected per patient and transported to the laboratory within 2 h of collection. Swabs were inoculated directly onto blood agar plates and incubated for 48 h before being examined. Latex coagulase testing was performed on all *Staphylococcus*-like colonies. Cefoxitin and trehalose mannitol salt (TMS) media were added to plates found to be beta-hemolytic, catalase-positive, and latex positive. Beta-hemolytic, catalase-positive, latex-negative plates also received TMS and additionally were tested with pyrrolidonyl arylamidase (PYR) for coagulase-negative *Staphylococcus*. Negative coagulase test plates were re-incubated and subsequently retested.

Acidic, yellow colonies indicated the presence of SA on the TMS plates. In plates with added Cefoxitin, SA colonies were classified as methicillin-resistant (MRSA) if the cefoxitin zone was ≤21 mm, and methicillin-susceptible (MSSA) if the zone was ≥22 mm. Patients found to be carriers of either MSSA or MRSA were treated with nasal mupirocin.

Data from the electronic record were collected on patients fitting the inclusion criteria. Study personnel, as approved by the IRB, collected the necessary data for the study via chart review. The data analysis consisted of a percentage analysis of the prevalence of positive MRSA patients.

## Results

A total of 400 patient charts were reviewed for MRSA and SA positivity, 61% were male and 39% were female. The mean age was 58 years (Table 1).

**Table 1:** **Patient population demographics with mean values**.

Characteristics	*N* (%)
**CHARACTERISTIC OF PATIENTS**	
**Sex**
Female	157 (39)
Male	243 (61)
**Pre-hospitalizing**
0	296 (74)
1	72 (18)
2	23 (6)
3	7 (2)
4	1 (1)
6	1 (1)
BMI [mean(SD)]	30 (7.5)
Age [mean(SD)]	58 (15.3)

Of the 400 total patients, 17 were found to be colonized with MRSA (4.25%). This corresponds to an MRSA prevalence of42 per 1000 patients with a standard deviation of 4.03. The 95% confidence interval for the population rate of MRSA is 0.0267–0.067.

**Figure 1:**
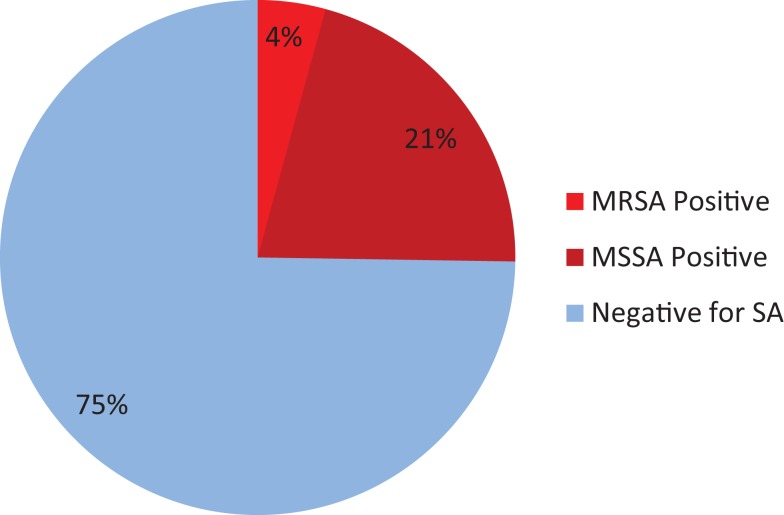
**Sample prevalence of *Staphylococcus aureus***.

In the same 400 patients, 101 were found to be positive for SA colonies, so the sampled percentage of SA-positive patients was 101/400 or 25.25%. This corresponds to SA prevalence of 250 per 1000 patients, with a standard deviation of 8.68. The 95% confidence interval for the population rate of SA is 0.2124–0.2973 (Figure 1).

The data were analyzed using Statistical Analysis Software, version 9.3 (SAS Institute Inc., Cary, NC, USA). Prior to analysis, the data were examined for outliers; no extreme values were found. Descriptive statistics are reported as mean ± SE and median (range) or total number and percentage. Binomial proportions and confidence intervals were provided for both MRSA and SA populations. Logistic regression analysis was conducted to determine the relationship between the main outcome (MRSA) and the other covariates including age, sex, BMI, and previous hospitalization (Table 2). Univariate logistical regression analysis was performed before multivariable analysis for each of the covariates, which were included in the full model if the covariate was significant (*p* < 0.20 at the 0.05 α level) in a two-sided test.

**Table 2:** **Statistical correlations between patient characteristics and MRSA positivity**.

Outcome	Characteristics	*P*-value
**ASSOCIATION BETWEEN MRSA AND PATIENT CHARACTERISTICS**		
MRSA	Age	0.196
	Sex	0.959
	BMI	0.968
	Pre-hospitalizing	0.214

### Limitations

This study was limited by a small sample size from a single institution of patients from similar geographic origin. Only the nares were tested for MRSA and SA colonies, which may have allowed for patients harboring extra-nasal colonies to appear as false-negative. The confidence intervals MRSA prevalence had a wide margin of error, suggesting the need for further study.

## Discussion

The prevalence of MRSA and SA in patients undergoing preoperative assessment for cardiothoracic and neurological surgery in Columbus, OH was analyzed. There were no significant differences between MRSA-positive and negative patient outcomes within the study, and no significant association between MRSA and patient characteristics was found.

This study provides insight on the extent of MRSA and SA colonization in a patient population particularly vulnerable to soft-tissue infection due to body-cavity exposure and extensive wound healing. In the sample population, 4.25% of patients tested positive for MRSA and 25.25% tested positive for SA. Confidence intervals were used to estimate the total population rate of MRSA and SA. This analysis accounts for the small sample size relative to the population size of cardiothoracic and neurological surgical patients.

The 95% confidence interval of MRSA carriers is 2.67–6.7% of cardiothoracic and neurological surgical patients. Likewise, the 95% confidence interval of SA carriers is 21.24–29.73%.

Compared to several other patient populations, preoperative cardiothoracic and neurological surgical patients have lower MRSA prevalence and, therefore, may be less likely to introduce community-acquired MRSA into hospitals. Populations with lower reported MRSA rates include pediatric healthcare workers and patients, as well as those in outpatient primary care clinics (12, 27, 28). Previous studies have reported cardiothoracic and neurological surgical patients to be at a lower risk for MRSA colonization than most other patient demographics including general surgery patients, emergency ward patients, and nursing home populations (22). Our study supported these previous reports.

Alone, these rates question the need for preoperative screening of cardiothoracic and neurological surgical patients. However, patients undergoing cardiothoracic and neurological surgeries are at a unique risk of MRSA-related SSIs when compared to patients undergoing orthopedic, otolaryngologic, gastrointestinal, and colorectal procedures. If screening criteria include the relative risk of SSI among MRSA carriers, then patients undergoing cardiothoracic and neurological surgery may be recommended for consideration in the development of targeted screening protocols.

## Conclusion

Improved carrier surveillance is an important aspect of limiting the introduction and transmission of MRSA in healthcare environments. Targeted screening allows carriers to be treated and also provides valuable epidemiological data to inform screening protocols. The current analysis indicates the preoperative prevalence of MRSA and SA in a sample of cardiothoracic and neurological surgical patients: 4.25% presented with MRSA and 25.25% with SA. According to prior studies, cardiothoracic and neurological surgical patients are at high risk for surgical-site infection, making preoperative assessment and treatment of these patients a valuable prevention of postoperative complications.

## Conflict of Interest Statement

The authors declare that the research was conducted in the absence of any commercial or financial relationships that could be construed as a potential conflict of interest.
